# Mapping Pituitary Neuroendocrine Tumors: An Annotated MRI Dataset Profiling Tumor and Carotid Characteristics

**DOI:** 10.1038/s41597-024-04218-8

**Published:** 2025-01-15

**Authors:** A. S. Pandit, A. Keenlyside, D. Z. Khan, G. Reischer, M. A. Kamal, N. Yoh, Z. Jaunmuktane, A. Borg, N. L. Dorward, S. E. Baldeweg, I. Davagnanam, H. Hyare, P. Nachev, H. J. Marcus

**Affiliations:** 1 High-Dimensional Neurology, Queen Square Institute of Neurology, University College London, London, UK; 2 Victor Horsley Department of Neurosurgery, National Hospital for Neurology and Neurosurgery, London, UK; 3 Wellcome/EPSRC Centre for Interventional and Surgical Sciences, University College London, London, UK; 4 Department of Neurosurgery, Columbia University, New York, USA; 5 Department of Neuropathology, National Hospital for Neurology and Neurosurgery, London, UK; 6 Department of Diabetes and Endocrinology, University College London Hospital, London, UK; 7 Centre for Obesity & Metabolism, Department of Experimental & Translational Medicine, Division of Medicine, University College London, London, UK; 8 Lysholm Department of Neuroradiology, National Hospital for Neurology and Neurosurgery, London, UK; 9 Department of Brain Repair and Rehabilitation, Queen Square Institute of Neurology, University College London, London, UK

**Keywords:** Pituitary diseases, CNS cancer, Pituitary diseases

## Abstract

Pituitary neuroendocrine tumors remain one of the most common intracranial tumors. While radiomic research related to pituitary tumors is progressing, public data sets for external validation remain scarce. We introduce an open dataset comprising high-resolution T1 contrast-enhanced MR scans of 136 patients with pituitary tumors, annotated for tumor segmentation and accompanied by clinical, radiological and pathological metadata. This diverse dataset captures variations in tumor size, location, and pathological activity, essential for understanding this complex condition. Expert annotations of both the tumor and adjacent carotid arteries ensure precise delineation, facilitating the development of automated segmentation algorithms. Our initiative addresses the need for standardized data in pituitary oncology, fosters collaboration and innovation, and enables the development and benchmarking of workflows that utilize pituitary radiomics for treatment planning and outcome prediction.

## Background & Summary

Pituitary neuroendocrine tumors are common lesions found in the sella and parasellar region, comprising about 15% of all intracranial tumors^1^. Pituitary tumors are categorized based on size, with macroadenomas exceeding 10 mm in diameter and microadenomas being smaller. Additionally, they are classified as functioning (FPAs) if they exhibit significant hormone activity, and as non-functioning (NFPAs) if they do not. Despite their classification as benign neoplasms, they can exhibit aggressive local growth and can result in systemic morbidity^2^. The challenges of diagnosing and accurately localizing these tumors, especially microadenomas, critically impact both the surgical approach and the prognosis^3^.

The management of patients with pituitary adenomas relies on a thorough assessment of their clinical and endocrine features. In recent years, tumor radiology has gained importance in guiding therapeutic decisions. Magnetic resonance imaging (MRI) remains the primary imaging modality for assessing pituitary gland abnormalities. It allows for the evaluation of a tumor’s volume and its spatial relationship with critical anatomical structures including the optic chiasm, cavernous sinus, and carotid arteries—key considerations for surgical planning. Moreover, radiomic profiling assists in predicting essential tumor properties such as consistency, invasiveness, hormonal activity, histopathological features, and likelihood of recurrence post-surgery^4^.

Underpinning radiomic analysis is the need for large-scale high-quality neuroimaging, with accurate lesion segmentation. However, few studies adopt the standards recommended for radiomic research^5^ and fewer still have pituitary data repositories available for public access. Indeed, large-scale data-sharing is necessary for the validation and full potential that radiomics represents. Additionally, significant variability across different scanner types, imaging sequences, and pre-processing methods underscores the importance of utilizing external, multi-vendor datasets to ensure robustness and generalizability of radiomic analyses^6^.

Pituitary MR imaging presents distinct challenges stemming from variations in scanning protocols across centers. Pituitary scans are often non-isotropic, limited to coronal and/or sagittal planes with a limited field of view and may lack T2-weighted signal information. This hampers inter-subject registration and limits the acquisition of certain radiomic features. Historical approaches to pituitary tumor annotation have demonstrated considerable variability, likely exacerbated by difficulties in distinguishing the gland from the tumor^7^. Previous radiomic analyses have also neglected proximity to critical neurovascular structures such as the carotid arteries, which significantly influences surgical outcomes.

To address these challenges, we present an isotropic, sub-millimeter voxel-sampled MR dataset comprising 136 patients. This comprehensive dataset includes whole-brain T1-weighted contrast-enhanced (T1w-CE) images, annotated to mark pituitary tumors and bilateral carotid arteries. Additionally, co-registered axial T2-weighted (T2w) images with matching voxel sizes are provided for the majority of cases.

## Methods

### Ethics and guidelines

This use of data from this cohort including anonymous data sharing was approved by the South-West regional Frenchay Research Ethics Committee (IRAS 271696). This repository follows, where relevant, the FAIR guidelines for data sharing^8^ and the radiomic quality score with respect to the source imaging data^5,7,8^.

### Patients

A review of our institutional database (a large tertiary neurosciences center with a high volume of pituitary referrals) was conducted to identify a series of all adult patients between July 2021 and December 2023. Patients meeting the following eligibility criteria were selected: (1) diagnosis of pituitary adenoma (2) high spatial resolution T1w-CE brain MRI performed prior to surgery. Patients who were having an operation for tumor recurrence or any pathology other than adenoma were excluded. All patients had been screened in the pituitary MDT with a consensus agreement for surgery made by attending neurosurgeons, endocrinologists and radiologists based on clinical and imaging information. Patients contemporaneously provide their consent for use of their data.

### Clinical and pathological information

Basic demographics (age, sex), clinical phenotype (non-functioning, Cushing’s disease, acromegaly, prolactinoma) were collected from a prospectively maintained surgical database. Histopathological data was obtained via pathology reports detailing cell lineage, cell type and Ki67 proliferative index according to WHO 2023 CNS tumor classifications.

The demographics of the cohort have been summarized in Table 1.

**Table 1:** Cohort demographic, endocrine, histopathological and radiological parameters.

	Characteristic	Value
Demographics	Mean age (range)	52.3 years (17 - 86)
	Female: male	66: 70
Endocrine	Functional status	Functional = 50 (Acromegaly = 22 Cushing’s disease = 16 Prolactinoma = 12) Non-functional = 85 N/A = 1
	Macro: micro	114: 22
Histopathology	Ki-67	Low / < 3 = 105 3–5 = 18 6+ = 3 N/A or negative stain = 10
	Cell type	Gonadotroph = 65 Corticotroph = 19 Somatotroph = 15 Lactotroph = 10 Mammosomatroph = 6 Thyrotroph = 2 Mixed = 2 Negative = 16
	Cell lineage	SF1 = 66 PIT-1 = 33 TPIT = 19 Negative/null = 16 Mixed = 2
Radiomics	Mean tumor volume (mm^3^) [range]	6126 [200–57000]

### Imaging

**Fig. 1:**
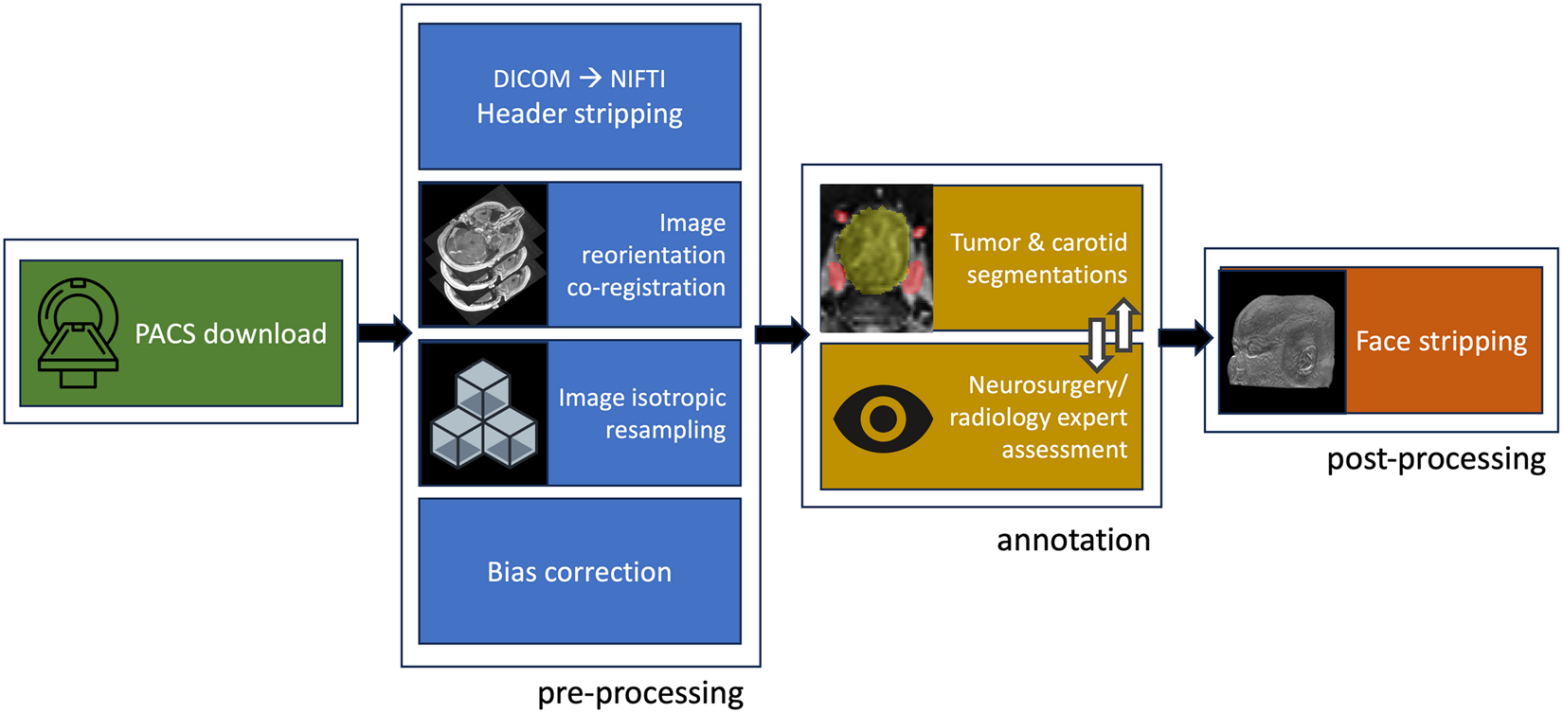
Processing pipeline for pituitary imaging data.

The salient steps involved in acquisition and processing of the pituitary imaging data have been outlined in Fig. 1.

### Scanning protocol

All patients had high spatial resolution T1w-CE sequences used for intraoperative neuronavigation compatible with the Medtronic Stealth 8® navigation system. These scans were generally performed internally of which the majority were performed 1-2 days prior to surgery. The original voxel dimensions for the Stealth sequence were 0.488 mm × 0.488 mm and 1.5 mm slice thickness. These scans were obtained on a Siemens Skyra or Prisma scanner at 3 T, with 512 × 512 × 114 matrix dimensions. The majority of patients also had a conventional T2w whole-brain turbo-spin echo (TSE) sequence performed. These were obtained on either a 1.5 or 3 T scanners with a range of matrix dimensions. A minority of patients had T2w imaging in pituitary-specific views. Each patient’s individual scanning information has been outlined in detail in the repository spreadsheet.

#### Pre-processing

Scans were downloaded from the institution’s PACS system in DICOM (Digital Imaging and Communications in Medicine) format. They were converted to NifTI format using the dcm2niix package^9^, at which point the imaging header data which includes patient identifiable information were stripped. All scans were reformatted using the *fslorient2std* function and then bias corrected using FMRIB’s Automated Segmentation Tool (FAST)^10^, both part of the FMRIB software library (FSL). T2-weighted images were co-registered to its T1W-CE counterpart using a 6 degree of freedom linear transformation applying a tri-linear interpolation and normalized mutual information cost function using FLIRT (FMRIB’s Linear Image Registration Tool). Image segmentation was then performed using these scans. All images were then resampled to 0.5 × 0.5 × 0.5 mm voxel sizes to ensure isotropy. We intentionally avoided cohort signal intensity normalization as (1) this is a step that can easily be performed by the user if they wish, (2) may significantly reduce the variance within the dataset and (3) was unlikely to affect the annotation process.

#### Post-processing

Following image annotation, images were then face stripped using cropping tools to ensure patient anonymity.

## Data Records

Imaging and associated clinical and pathological metadata are available on Figshare^11^. Pre-processed T1-weighted, contrast-enhanced MR images have been stored in NifTI format, alongside manual/semi-automated segmentations of (1) the pituitary tumor-gland complex and (2) bilateral intracranial carotid arteries. Co-registered T2-weighted imaging, if available, has also been uploaded. Therefore, for each patient, there are 3 or 4 imaging files with the following endings, prefixed by their anonymised identifier: [pid]_T1.nii.gz, [pid]_T2.nii.gz, [pid]_tumour.nii.gz, [pid]_carotids.nii.gz. One supplementary excel file containing: (1) relevant clinical metadata, (2) scanning parameters (3) neuropathological information and (4) basic radiomic data.

## Technical Validation

In developing this repository, rigorous measures were implemented to uphold the quality and validity of the dataset. Each MR image was inspected at several time points as part of the processing framework (Fig. 1). The initial inspection occurred at the point of conversion from DICOM to NiFTI. Subsequent evaluations were conducted after image pre-processing and intra-subject registration, followed by a final check after face-stripping. Accordingly, patients were excluded if images were degraded by motion or other scanning artifacts, if registration or pre-processing had failed, or if face-stripping appeared insufficient.

### Image segmentation

Image segmentation was performed using ITK-Snap^12^, a widely used image annotation tool for neurosurgical lesions^13^ including pituitary tumors^14^ which has both manual and semi-automated segmentation functions.

To segment tumors, a region of interest on isovolumetric T1 encompassing the tumor was supersampled by a factor of 2 in ITK-snap. Within this region, an intensity window was set to isolate the greatest extent of the tumor soft tissue from the carotids and surrounding CSF. Then a ‘region competitive snake’ segmentation was initialized using manually placed spheres covering the tumor and iteratively reduced to fit the tumor volume (balloon force of 0.1 and curvature force of 0.9).

Carotid segmentations were approached similarly, however using a lower limit intensity threshold, to isolate the typically higher intensity carotids. These were initialized using a string of smaller spheres to cover the desired length of the carotids and iterated over, using the same region’s competitive parameters. For both carotids and tumors, manual adjustments were made using both T1- and T2- weighted images where required. Segmentations were smoothed using a full width at half maximum (FWHM) Gaussian kernel and eroded, followed by a clustering algorithm to remove stray voxels.

### Segmentation quality

Annotations were performed by neurosurgical residents and students familiar to the pituitary operative workflow and who were pre-trained on a dataset not used for this repository. Segmentation quality was then assessed by both an attending board-certified neurosurgeon with a subspeciality interest in pituitary and anterior skull base tumors and attending board-certified neuroradiologists with subspeciality interests in neuro-oncology and anterior skull base. Therefore, in total, each patient’s images were reviewed by a neurosurgical resident at least four times and by subject matter experts twice.

For tumors and carotid arteries, the quality of annotations was assessed using criteria described in Tables 2, 3 respectively. If any of the checkpoints were not met, the segmentation was edited before re-assessment.

**Table 2:** Criteria used to assess pituitary tumor segmentation quality.

Component	Checkpoint
Superior boundary	- identify arachnoid boundary separating sella contents
	- assess proximity and delineation of the tumor annotation to the hypophysis, optic chiasm (generally non-enhancing), third ventricle, and gyri rectii
Antero-inferior boundary	- assess proximity and delineation of the tumor annotation to the sella turcica (if not eroded), sphenoid sinus and clivus. If available, correlate with fine-cut bony CT
Posterior boundary	- assess proximity and delineation of the tumor annotation to the clivus and, if eroded, the pre-pontine and interpeduncular cisterns. If available, correlate with fine-cut bony CT
Lateral boundaries	- inspect for effacement of the cavernous sinus (check the median inter-carotid line in coronal section^15^) and if laterally displacing, observe for contrast differences between the tumor and cavernous sinus wall to ensure correct annotation
Contents	- if tumor extends to surrounding dura - including the dural-tumor interface
	- if the pituitary gland is distinct as for most microadenomas, check annotation only covers the tumor, else the complete tumor-gland complex
	- ensure any tumor cystic components are within the tumor annotation

**Table 3:** Criteria used to assess internal carotid artery segmentation quality.

Component	Checkpoint
Segments	- ensure annotation covers all relevant segments of the internal carotid arteries: cavernous, supraclinoid and ophthalmic. If available, correlate with T2-weighted imaging
Boundaries	- check and ensure no areas of overlap between tumor and arterial annotation

## Usage Notes

The provided dataset is valuable for pituitary tumor radiomics, offering opportunities for training and testing algorithms in lesion segmentation and treatment evaluation. Additionally, it serves as a versatile resource for translational research, enabling investigations into pathological correlations, population trends, and hypothesis validation. By ensuring the entirety of the tumor is captured and also its spatial relationship with the internal carotid arteries, this method of segmentation was felt to be most relevant for neuronavigation and surgical application. As scan resolution and image processing improve and the database is updated, the accuracy of the segmentation will iteratively increase facilitating closer approximation to the true tumor ground truth.

## Data Availability

The repository has been made available in whole, with associated pre-processing scripts without undue reservation on Figshare^11^.
